# Study Protocol: Adjuvant Holmium-166 Radioembolization After Radiofrequency Ablation in Early-Stage Hepatocellular Carcinoma Patients—A Dose-Finding Study (HORA EST HCC Trial)

**DOI:** 10.1007/s00270-022-03162-7

**Published:** 2022-05-26

**Authors:** Pim Hendriks, Daphne D. D. Rietbergen, Arian R. van Erkel, Minneke J. Coenraad, Mark J. Arntz, Roel J. Bennink, Andries E. Braat, A. Stijn L. P. Crobach, Otto M. van Delden, Tom van der Hulle, Heinz-Josef Klümpen, Rutger W. van der Meer, J. Frank W. Nijsen, Carla S. P. van Rijswijk, Joey Roosen, Bastian N. Ruijter, Frits Smit, Mette K. Stam, R. Bart Takkenberg, Maarten E. Tushuizen, Floris H. P. van Velden, Lioe-Fee de Geus-Oei, Mark C. Burgmans, Dutch Hepatocellular Cholangiocarcinoma Group

**Affiliations:** 1 Department of Radiology, Leiden University Medical Center, P.O. Box 9600, 2300 RC Leiden, The Netherlands; 2 Department of Gastroenterology and Hepatology, Leiden University Medical Center, Leiden, The Netherlands; 3 Department of Medical Imaging, Radboud University Medical Center, Nijmegen, The Netherlands; 4 Department of Radiology and Nuclear Medicine, Amsterdam University Medical Center, Location AMC, Amsterdam, The Netherlands; 5 Department of Surgery, Leiden University Medical Center, Leiden, The Netherlands; 6 Department of Pathology, Leiden University Medical Center, Leiden, The Netherlands; 7 Department of Medical Oncology, Leiden University Medical Center, Leiden, The Netherlands; 8 Department of Medical Oncology, Amsterdam University Medical Center, Location AMC, Amsterdam, The Netherlands; 9 Department of Gastroenterology and Hepatology, Amsterdam University Medical Center, Location AMC, Amsterdam, The Netherlands

**Keywords:** Hepatocellular carcinoma, Early-stage HCC, Thermal ablation, Radiofrequency ablation, Radioembolization, Holmium-166, TARE

## Abstract

**Purpose:**

To investigate the biodistribution of holmium-166 microspheres (^166^Ho-MS) when administered after radiofrequency ablation (RFA) of early-stage hepatocellular carcinoma (HCC). The aim is to establish a perfused liver administration dose that results in a tumoricidal dose of holmium-166 on the hyperaemic zone around the ablation necrosis (i.e. target volume).

**Materials and Methods:**

This is a multicentre, prospective, dose-escalation study in HCC patients with a solitary lesion 2–5 cm, or a maximum of 3 lesions of ≤ 3 cm each. The day after RFA patients undergo angiography and cone-beam CT (CBCT) with (super)selective infusion of technetium-99 m labelled microalbumin aggregates (^99m^Tc-MAA). The perfused liver volume is segmented from the CBCT and ^166^Ho-MS is administered to this treatment volume 5–10 days later. The dose of holmium-166 is escalated in a maximum of 3 patient cohorts (60 Gy, 90 Gy and 120 Gy) until the endpoint is reached. SPECT/CT is used to determine the biodistribution of holmium-166. The endpoint is met when a dose of ≥ 120 Gy has been reached on the target volume in 9/10 patients of a cohort. Secondary endpoints include toxicity, local recurrence, disease-free and overall survival.

**Discussion:**

This study aims to find the optimal administration dose of adjuvant radioembolization with ^166^Ho-MS after RFA. Ultimately, the goal is to bring the efficacy of thermal ablation up to par with surgical resection for early-stage HCC patients.

**Trial registration:**

Clinicaltrials.gov identifier: NCT03437382.

**Supplementary Information:**

The online version contains supplementary material available at 10.1007/s00270-022-03162-7.

## Introduction

Thermal ablation (TA) has proven to be an effective treatment for hepatocellular carcinoma (HCC) and it has become the treatment of first choice in solitary lesions up to 2 cm owing to its equal effectiveness and lower complication rate compared with surgical resection [1]. In patients with a preserved liver function and larger solitary, or up to 3 HCC lesions of ≤ 3 cm, surgical resection remains the preferred treatment modality [1, 2], as it yields a better oncological outcome [3–5]. Yet, surgical resection is often contraindicated due to liver cirrhosis with portal hypertension, deranged liver function, comorbidity or an unfavourable tumour localization [1].

Efforts to prevent tumour recurrence are key to improve the long-term prognosis of HCC patients treated with TA. Recent systematic reviews show that the chance of developing local tumour progression (LTP) is higher after TA compared to surgical resection, especially in the treatment of lesions > 3 cm [4, 5]. Causes for higher LTP rates in larger tumours are (a) insufficient heat generation or propagation at the peripheral parts of the tumour, (b) viable satellite nodules found in the direct proximity of the main tumour and (c) the ‘heat-sink effect’ near medium to large blood vessels. Regardless of the cause, local recurrence after TA is most commonly seen at the periphery of, or in close proximity to the main tumour [6].

External beam radiation therapy is widely used as an adjuvant therapy to surgery in different types of cancer, but is infrequently used to treat liver cancer, as the liver has a low tolerability to it and liver cirrhosis further reduces this tolerability [7, 8]. Preclinical studies identified potential benefits of combined radiofrequency ablation (RFA) and radiation-based therapy too [9–12]. Potential causes for synergy between RFA and radiation-based therapy include the sensitization of viable tumour cells to subsequent radiation owing to the increased oxygenation resulting from hyperaemia, like in hyperbaric radiotherapy [13]. Another possible synergetic result may be a radiation-induced inhibition of repair and recovery and increased free radical formation, as observed in animal tumour models with RFA and transarterial chemoembolization (TACE) [14]. Transarterial radioembolization (TARE) provides an alternative way of delivering adjuvant radiation therapy by means of radioactive microspheres that are administered selectively in the hepatic artery using a high tumour dose and a low toxicity to the healthy liver parenchyma [15, 16].

RFA induces hyperaemia in a marginal zone around the area of ablation necrosis [17]. This hyperaemic zone encompasses the area in which viable residual tumour cells or satellite nodules may reside. When TARE is administered shortly after RFA, it is hypothesized that the hyperaemia can be used to deliver a large amount of holmium-166 microspheres (^166^Ho-MS) to this marginal zone with the aim of decreasing the chance of LTP. The objective of this study is to find the necessary administrated dose of ^166^Ho-MS that yields a dose of ≥ 120 Gy to the hyperaemic zone (target volume).

## Methods

This is a multicentre, open-label, non-randomized, phase I dose-escalation study of the use of adjuvant TARE after RFA in HCC patients with a solitary lesion of 2–5 cm, or a maximum of 3 lesions of ≤ 3 cm each. Leiden University Medical Center is the sponsor of the study. The trial will be executed in 3 academic hospitals (see Supplementary Table 1).

### Eligibility Criteria

A full list of in- and exclusion criteria can be found in Table 1. Patients with BCLC early-stage HCC (A) are eligible if they have a solitary lesion of 2–5 cm or a maximum of 3 lesions of ≤ 3 cm each, and if surgical resection would not be the treatment of first choice as decided upon by the multidisciplinary tumour board. General contraindication criteria for RFA and TARE are used [1, 2]. Additional exclusion criteria where: a) a treatment volume (i.e. area exposed to radiation) exceeding 50% of the total liver volume and b) creatinine clearance rate < 30 mL/min.

**Table 1:** List of in- and exclusion criteria

Inclusion criteria	Exclusion criteria
Informed consent	Tumour location precluding percutaneous RFA
Age > 18 years	Treatment volume > 50% of total liver volume, based on CBCT images
Single HCC lesion with diameter of ≥ 2-5 cm or up to three lesions with each lesion measuring no more than 3 cm	Vascular tumour invasion or extrahepatic metastasis
HCC diagnosis is based on histology or non-invasive imaging criteria according to EORTC-EASL guidelines	Hemihepatectomy
Child Pugh A or B ≤ 7	Severe comorbidity (e.g. cardiovascular disease, diabetes with nephropathy, active infections)
(HCC-unrelated) ECOG performance status ≤ 2	Uncorrectable coagulopathy
Bilirubin < 2 mg/dL	Large arterio-portovenous shunt
ASAT < 5 × upper limit of normal	Previous radiotherapy to the liver
ALAT < 5 × upper limit of normal	Surgical hepatico-enterostomy
Thrombocytes ≥ 50 X 10^9/L	Hepatic resection with placement of surgical clips that may cause artefacts on MRI
	Incompetent/ mentally disabled
	Pregnancy, inadequate anticonception
	Lung shunt fraction > 20%
	Creatinine clearance < 30 mL/min/1.73m^2^

### Interventions

**Fig. 1:**
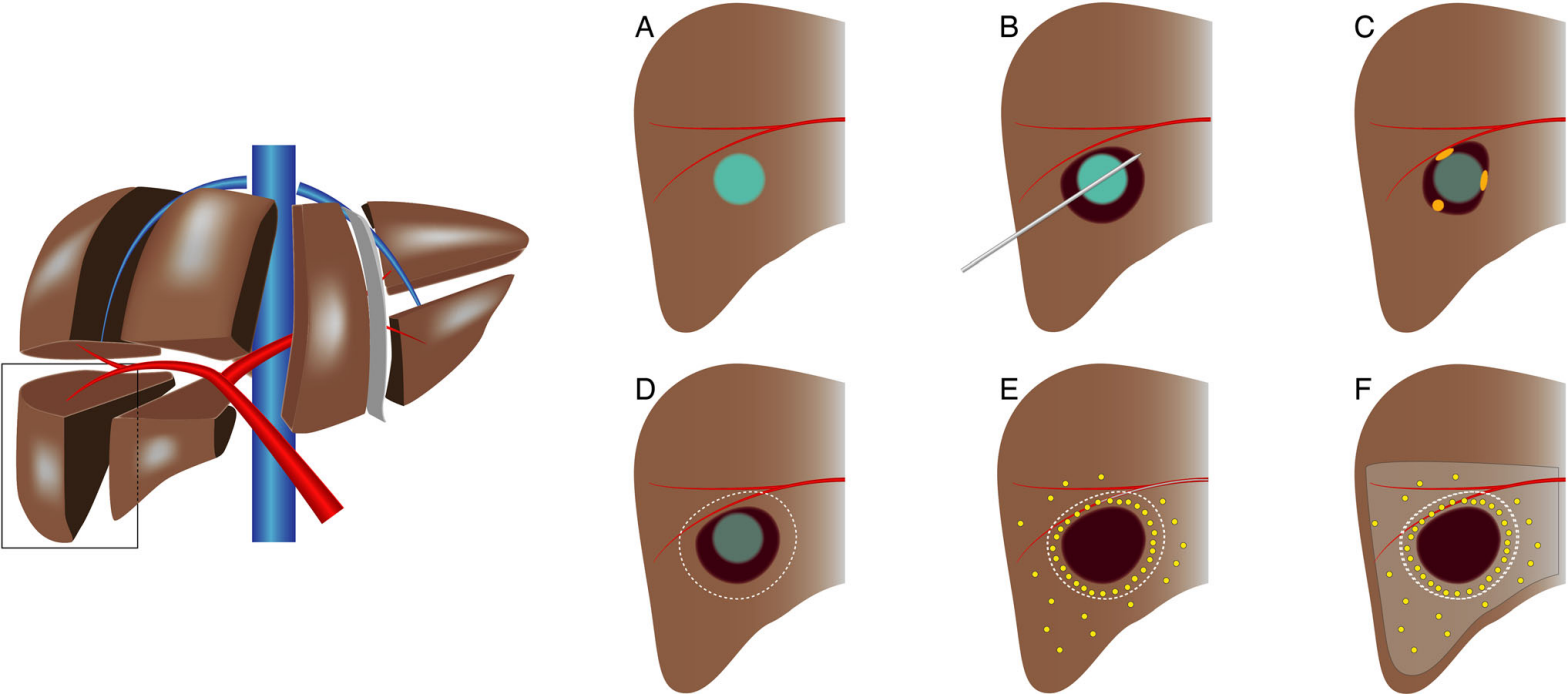
Schematic drawings of the study procedure. **A** HCC lesion of 2–5 cm. **B** Thermal ablation of HCC lesion. **C** Potential sites of LTP due to heat-sink effect, impaired heat propagation or satellite nodules. **D** Target zone for adjuvant TARE. **E** Deposition of ^166^Ho-MS with preferential flow of microspheres to the hyperaemic zone surrounding the ablation area. **F** Perfused liver volume after ^166^Ho-MS TARE (i.e. treatment volume)

A schematic overview of the study procedure can be found in Fig. 1. RFA is performed under general anaesthesia or deep sedation using single or three 3 or 4 cm exposed tip multi-electrode Cool-tip RFA probes with switch-control system (Medtronic Inc, Dublin, Ireland). A contrast-enhanced computed tomography (CECT) scan is performed on a 64-slice Aquilion CT-scanner (Canon, Tochigi, Japan) immediately after ablation and additional ablation is performed in the same session when residual tumour tissue is identified on this scan.

On the second day, angiography and administration of 150 MBq of ^99m^Technetium-labelled macro-albumin aggregate (^99m^Tc-MAA) are performed with a single photon emission/ computed tomography (SPECT/CT) scan directly after the procedure on a Symbia T6 or Symbia Intevo (Siemens Healthineers, Erlangen, Germany) or Discovery 670 Pro (GE Healthcare, Boston, Massachusetts, USA). Prior to injection a contrast-enhanced cone-beam CT (CBCT) is performed to verify the treatment volume, and potassium perchlorate was given to patients [18]. Hepatico-enteric anastomoses are coiled if necessary. Using a Progreat 2.4F or 2.7F microcatheter (Terumo Corporation, Tokyo, Japan), catheter position(s) is/are chosen as selectively as possible for ^99m^Tc-MAA-injection. Multiple catheter positions may be used to ensure adjuvant treatment of the entire hyperaemic zone(s) after ablation. The SPECT/CT scan is used to rule out lung shunting > 20%.

On day 5–10 after RFA, TARE with ^166^Ho-MS QuiremSpheres (Quirem Medical B.V., Deventer, The Netherlands) is performed. The administration activity of holmium-166 (AHo-166) is calculated using the following equation [19]:

$${A}_{Ho-166}=Perfused Liver Dose \left[\mathrm{Gy}\right]*{W}_{i} [\mathrm{kg}]*63 [\mathrm{MBq}/\mathrm{J}].$$

Depending of the cohort, patients are treated with 60, 90 or 120 Gy to the treated liver segments. The weight of the treated volume (*w*_i_) is determined by the treatment volume as segmented from the CBCT, determined using IntelliSpace software (Philips Healthcare, Eindhoven, The Netherlands), using an anticipated tissue density of 1.00 g/cm^3^. The catheter position for Ho-166 injection is verified by fluoroscopic and CBCT imaging prior to infusion.

Post-treatment SPECT/CT is performed the day after TARE for dosimetry purposes. Moreover, magnetic resonance imaging (MRI) is acquired between RFA and TARE, and after Ho-166 treatment. T2* sequences are acquired on a 1.5 T Ingenia MRI system (Philips Healthcare, Eindhoven, The Netherlands) or 3 T Magnetom Skyra (Siemens Healthineers, Erlangen, Germany) for post-treatment dosimetry purpose, by subtracting these scans, making use of the paramagnetic properties of Ho-166 [19–21].

**Fig. 2:**
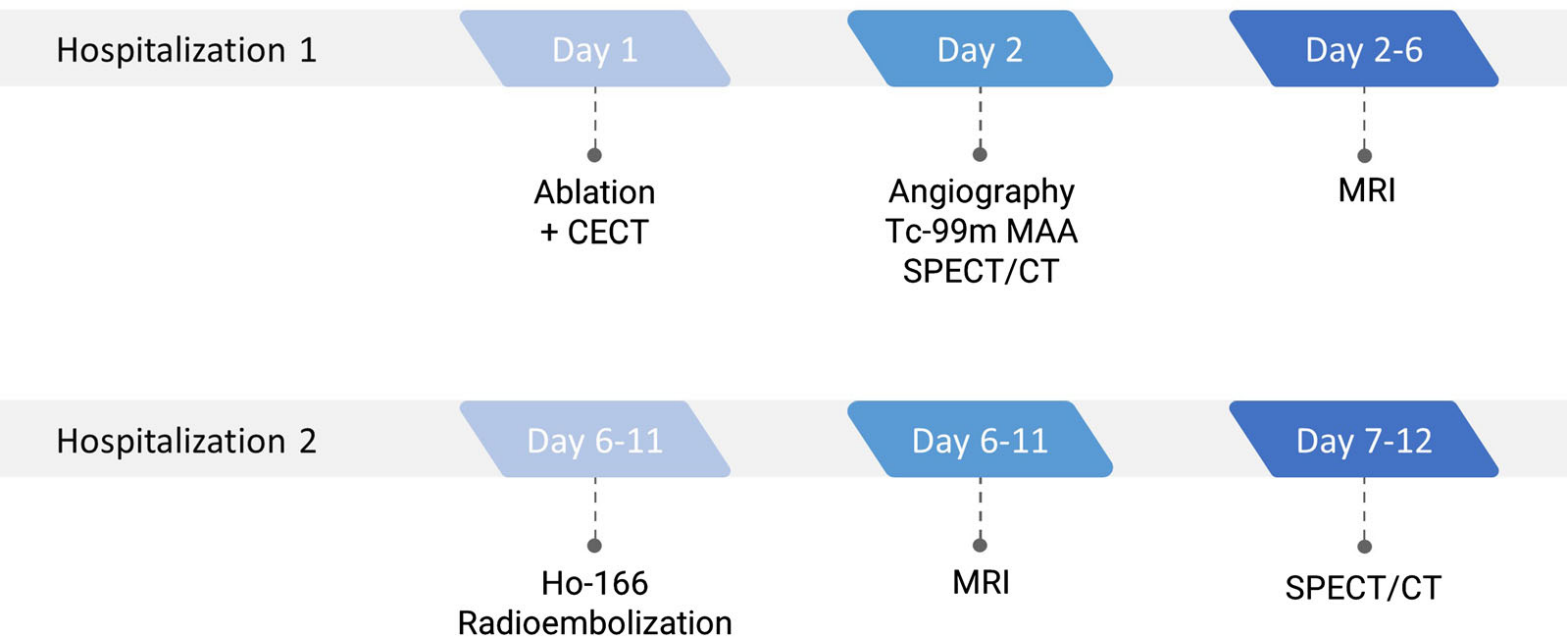
Participants timeline of the treatment period. After the angiography procedure and the acquisition of the Tc-99 m MAA SPECT/CT, the dose calculation was performed and ^166^Ho-MS were ordered

A participant’s timeline of the two hospitalizations can be found in Fig. 2.

### Follow-Up

Patients are followed for 12 months after treatment. The follow-up is performed according to regular HCC treatment regimen. Imaging follow-up will be performed by CECT or MRI at 6 weeks and 3 months after treatment and then every 3 months. Clinical assessment and biochemical liver function tests are performed at 2 weeks, 6 weeks and 3 months and continued synchronized with imaging. Adverse events will be categorized according to common terminology criteria for adverse events (CTCAE) 4.0 [22]. Serious adverse events will be immediately reported to the ethical board upon notification.

### Outcomes

Different small cohorts are exposed to 60 Gy, 90 Gy or 120 Gy to the treated liver volume. The primary endpoint of this study is to find the treatment volume dose that results in a dose of ≥ 120 Gy to the target volume in 9/10 patients, based on post-treatment SPECT scan. The hyperaemic zone encompassing the ablation necrosis (or necroses) is considered the target volume and generally anticipated to be a 1 cm rim around the ablation necrosis/necroses. Segmentation of the treatment and target volumes in the post-treatment SPECT scan is performed using Xeleris workstation version 4.0 (GE Healthcare, Boston, Massachusetts, USA). The study consists of a maximum of 3 cohorts (treatment volume doses of 60 Gy, 90 Gy and 120 Gy), depending on when the final endpoint is met. If the second patient within one cohort fails to meet ≥ 120 Gy on the target volume, the study endpoint of 9/10 patients fails and the cohort is closed. Consecutive patients are then treated with a higher dose as part of the following cohort.

Secondary endpoints include toxicity according to CTCAE 4.0, disease-free and overall survival.

### Sample Size

No sample size calculations were performed as this is a phase I feasibility study. A minimum of 10 patients will be recruited when ≥ 9/10 patients will meet the endpoint of 120 Gy to the target volume at a treatment dose volume dose of 60 Gy. A maximum of 30 patients will be recruited as there are maximally 3 cohorts in this study with a maximum of 10 patients in each cohort.

### Data

The obtained CBCT, SPECT/CT and MRI scans will be pseudonymized and stored in an encrypted folder accessible only to the PI and study coordinator. Pseudonymized patient baseline, study and follow-up data are stored in an encrypted database by Castor EDC (Castor, Amsterdam, The Netherlands). Data will be subject to data monitoring every year.

## Discussion

This is the first clinical trial in which TARE is investigated as adjuvant therapy after TA in patients with HCC. Advancements in tumour targeting, treatment planning and evaluation have led to increased efficacy of TA with clinical studies reporting local recurrence rates comparable to surgery even for tumours > 2 cm [23]. Nevertheless, in larger clinical trials these findings have not been confirmed and surgical resection remains the recommendation for solitary HCC lesions > 2 cm in the recently published update of the BCLC system [24]. The task that lies ahead for interventional radiologists is to bring the efficacy of thermal ablation up to par with that of surgical resection.

The combination of TA with either systemic therapy or transarterial therapy has been investigated in different studies. In the STORM trial, no difference was found in median recurrence free survival between patients treated with adjuvant sorafenib or placebo [25]. Currently, several trials combining thermal ablation with molecular or immuno-therapy are ongoing [26]. The most widely investigated combination therapy is that of TA and TACE. Superiority of TACE-RFA compared with RFA with respect to LTP after treatment of lesions > 2 cm was found in a recent meta-analysis [27]. Nevertheless, validation in a western cohort is lacking and it is not recommended in the European guidelines. Furthermore, there is a lack of consensus on how the two therapies are best combined with respect to sequence, interval and embolic agent [28].

Over recent years, radiation segmentectomy has received attention as an alternative to thermal ablation. In the LEGACY study, patients with a solitary HCC up to 8 cm were treated with a high dose of yttrium-90. The results of this trial were promising, since a high local control rate was found, which led to the acceptance of radiation segmentectomy as a treatment for patients that are not a candidate for resection or ablation [29]. Limitations of the LEGACY study are that this was a retrospective study and mean tumour size was only 2.7 cm. Prospective comparative studies are warranted before radiation segmentectomy can be further implemented in clinical practice.

Our study investigates the combination of TA and TARE. This is a first-in-man study to investigate the biodistribution of ^166^Ho-MS when administrated shortly after RFA. The data will be used in future prospective studies investigating the efficacy of combined thermal ablation and TARE, with the long-term objective to bring the efficacy of TA up to par with surgical resection for HCC > 2 cm.

With respect to TA, the current protocol only permits the use of RFA. Microwave ablation (MWA) may have technological advantages over RFA, but yet similar outcomes are found [30]. In order to minimize variability in technique and materials, it was chosen to perform all TA procedures with RFA and with the same system. Furthermore, (pre-)clinical work on the combination of TA and radionuclide therapy has so far only been performed with RFA [9–12].

In this study, TARE is used as an adjuvant rather than as a neoadjuvant therapy. In this way, TARE can be used to target the marginal zone that corresponds to the area where LTP is most commonly seen after TA. When TARE is performed shortly after the ablation, a preferential flow of ^166^Ho-MS to the hyperaemic volume is expected. This principle has also been utilized in studies investigating TA with adjuvant TACE the next day. In our study, the interval between TA and TARE ranges between 5 and 10 days, which is mainly due to logistical reasons. Every patient receives an individualized treatment dose and the microspheres need to be prepared in a nuclear facility prior to administration. There is sufficient evidence though, that the aforementioned hyperaemia persists during the first weeks and sometimes even months [31].

In this study, ^166^Ho-MS were used rather than yttrium-90. Holmium-166 offers specific advantages as it emits gamma radiation at 81 keV besides the therapeutical beta particles, allowing for quantitative SPECT. Moreover, due to its paramagnetic properties, post-treatment dosimetry can also be performed using MRI. Data from the HEPAR 1 study were used to determine the dose for the first patient cohort, i.e. patients treated with a treatment volume dose of 60 Gy [32]. In the HEPAR 1 study, an administrated dose of 60 Gy was established as the maximal tolerated for patients with multiple liver metastases. A dose escalation to a maximum of 120 Gy is expected to be safe, as no more than 50% of the non-tumorous liver parenchyma will be exposed to radiation and only patients with a preserved liver function are allowed to participate in the study. The treatment volume is calculated using CBCT images as these provide the best insight into the vascular territories of tumour-feeding arteries. No data were available on the dose–response relationship for holmium-166 radioembolization at the time the study was designed. A target dose of 120 Gy was chosen in close consultation with Quirem Medical B.V. (producer of ^166^Ho-MS). Although well aware of the potential differences in radiobiology between yttrium-90 and holmium-166, this was based on earlier ^166^Ho-MS cases and based on yttrium-90 therapy standards, prior to more recently published dose–response evaluation studies. Several studies investigating ^166^Ho-MS are currently ongoing, including the HEPAR PRIMARY trial. Those studies are expected to provide further insight into the dose–response relationship of holmium-166.

As an exploratory endpoint, MRI-based dosimetry will be performed. Yet, to determine the absorbed dose on the target volume and to determine the primary endpoint of the study, SPECT/CT imaging will be used. SPECT/CT will be able to give an estimate of the absorbed radiation dose, but due to the limited spatial resolution it will be difficult to determine the precise border of the target volume. The thickness of the hyperaemic zone, i.e. target volume, will be measured on the post-ablation diagnostic CT and CBCT images. In general, a rim of 1 cm around the ablation zone will be considered as the target volume as most satellite tumours reside within 1 cm from the primary tumour [33].

The TARE work-up is performed with ^99m^Tc-MAA in this study whereas Ho-166 specific work-up could also be performed with Ho-166 scout dose [34]. At the time of the initial study design, Ho-166 scout dose was not commercially available yet. Moreover, since the work-up was only used for ruling out high lung-shunt fractions rather than partition model-based dosing, ^99m^Tc-MAA was deemed sufficient.

The goal of the current trial is to study the feasibility and dosimetry of TARE as adjuvant treatment after TA for HCC patients. In this trial, all early-stage patients with a solitary tumour of 2–5 cm or a maximum of 3 tumours of ≤ 3 cm each can be included, while this study focuses on the proof of concept of combining the treatments. In future trials, further specification of patient characteristics should be defined to identify which patients potentially benefit most from this treatment combination. Moreover, these trials should reveal the potential clinical benefit of this new treatment combination in terms of disease-free and overall survival.

## Supplementary Information

Below is the link to the electronic supplementary material.

Supplementary file1 (DOCX 13 kb)
