# Supplementary material for: Study Protocol: Adjuvant Holmium-166 Radioembolization After Radiofrequency Ablation in Early-Stage Hepatocellular Carcinoma Patients—A Dose-Finding Study (HORA EST HCC Trial)
Source: Cardiovasc Intervent Radiol. 2022 May 26;45(8):1057–63. doi: 10.1007/s00270-022-03162-7 (PMC9307549; doi:10.1007/s00270-022-03162-7)
Supplement: Supplementary file 1 — Supplementary file1 (DOCX 13 kb) [file 270_2022_3162_MOESM1_ESM.docx]

**Supplementary table 1** Overview of the institutions involved in the HORA EST HCC trial and their role.

| **Institutions** | **Role in the study** |
| --- | --- |
| Leiden University Medical Center | Sponsor, inclusion center, data management and providing the principal investigator |
| Radboud University Medical Center | External inclusion center |
| Amsterdam University Medical Center | External inclusion center |
| Health~Holland | Funding |
| Dutch association of stomach liver and bowel disease MLDS | Funding |
| Quirem Medical B.V. | Sponsoring |
| Medtronic | Sponsoring |
| Dutch Hepatocellular and Cholangiocarcinoma Group | Endorsement and advisor |
| Dutch association of liver patients NLV | Endorsement and advisor |
|  |  |
